# Computer Simulation of Tooth Wear in Giant Pandas (*Ailuropoda melanoleuca*): Quantitative Predictions for Captive Health Management

**DOI:** 10.3390/ani16142273

**Published:** 2026-07-22

**Authors:** Fuhao Chen, Yichao Yang, Xiaogang Shi, Xixuan Zhang, Yue Zou, Ti Li, Panyan Yang, Yuanyuan Qu, Hongqiang Lin, Jiangwei Xiang, Zhijun Zhong, Guangneng Peng, Kun Zhang, Quan Mo, Haifeng Liu, Chengdong Wang, Ziyao Zhou

**Affiliations:** 1 College of Veterinary Medicine, Sichuan Agricultural University, Chengdu 611130, China; vetchenfh@163.com (F.C.);; 2 Chongqing Fengdu Industrial Development Group Co., Ltd., Chongqing 408200, China; 3 Sichuan Wolong National Natural Reserve Administration Bureau, Wenchuan 623006, China; 4 China Conservation and Research Center for the Giant Panda, Key Laboratory of SFGA on the Giant Panda, Chengdu 611130, China

**Keywords:** the giant panda, tooth wear, computer simulation, captive management, animal welfare

## Abstract

Tooth wear is a major health and welfare issue for captive giant pandas (*Ailuropoda melanoleuca*), but quantitative data on wear progression across different tooth types and chewing behaviors are lacking. In this study, a two-dimensional computer simulation (Processing, Java-based) was developed to model wear of giant panda canine, second premolar, and second molar teeth under two bamboo-chewing orientations (horizontal vs. vertical). Species-specific enamel hardness (344 HV), bite forces (canine: 1030 N; premolar: 1142 N; molar: 2005 N), and a stochastic chewing cycle were incorporated into the simulation. The results showed that canine wear was faster during horizontal chewing (45.68 pixel layers/100 cycles) than during vertical chewing (36.76 pixel layers/100 cycles), while premolar and molar wear were faster during vertical chewing (34.24 and 51.52 pixel layers/100 cycles, respectively) compared to horizontal chewing (10.28 and 39.40 pixel layers/100 cycles). Molars exhibited the highest overall wear rate. The predicted functional lifespan was estimated to be 47~55 years for canines; 19~34 years for premolars; and 23~29 years for molars. This study provides the first quantitative, cycle-by-cycle wear progression model for giant panda teeth.

## Simple Summary

Giant pandas spend most of their day eating bamboo, which gradually wears down their teeth. Severe tooth wear can cause pain, difficulty eating, and shortened lifespan. We developed a computer simulation to predict how quickly different tooth types—canines, premolars, and molars—wear when pandas chew bamboo in different directions (horizontal or vertical). Our results show that molars wear the fastest, followed by premolars, while canines wear the slowest. Under natural chewing conditions, premolars and molars are predicted to lose function by approximately 19–23 years of age, whereas canines may last beyond 45 years. Chewing direction also matters: canines wear more when bamboo is held sideways (horizontal), while cheek teeth wear more when bamboo is chewed lengthwise (vertical). These findings might help zoo managers and veterinarians plan dental check-ups and adjust diets for older pandas, and indicate that they should focus on premolars and molars as the most vulnerable teeth.

## 1. Introduction

The giant panda (*Ailuropoda melanoleuca*) is a global icon of wildlife conservation. Its survival is challenged by habitat loss, low reproductive rates, and age-related health issues [1]. Among these, dental health is a critical but often overlooked factor influencing individual fitness, lifespan, and population viability [2,3]. Although phylogenetically classified within Carnivora, the giant panda has evolved a specialized bamboo-dominated diet (with bamboo accounting for ~99% of its daily food intake), including high-fiber, abrasive species such as cold-arrow bamboo (*Bashania fangiana*) [4]. To meet basal energy requirements, an adult panda consumes 12–38 kg of bamboo daily [5], subjecting its dentition to repetitive, high-intensity mechanical loading.

Many grazing herbivores evolve high-crowned hypsodont teeth to cope with long-term abrasive plant feeding; in contrast, giant pandas retain short brachydont teeth inherited from their carnivorous ancestors, which is a unique morphological contradiction. Typical browsing herbivores also present low-crowned dentition, which differs from the tooth structure of giant pandas [2,3].The broad, irregular square-shaped occlusal surface of the giant panda’s molars efficiently grinds hard bamboo culms into readily digestible fragments. The expansive and uneven occlusal surface provides an ideal mechanical structure for comminuting tough bamboo stems [6].This morphological “paradox”—carnivore-shaped teeth used for an abrasive herbivorous diet—creates a unique scenario of tooth wear that differs from both typical carnivores and typical herbivores [7]. Notably, the temporomandibular joint (TMJ) of the giant panda permits lateral movement of the mandible. This lateral movement essentially represents a “postural shift” of the jaw during mastication—a transition from opening-closing motion to lateral sliding—enabling different teeth (premolars versus molars) to function at different stages of bamboo processing [6]. Clinical observations of the authors have revealed that giant pandas employ different teeth and continuously vary their chewing directions during bamboo consumption (Supplemental Material, Video S1). Bamboo cell walls are rich in naturally abrasive phytoliths, and their prolonged and repeated mastication continuously disrupts the specialized enamel microstructure of giant pandas, substantially compromising dental integrity and masticatory efficiency. Moreover, the species engages in 12–16 h of high intensity chewing activity daily (they spend ~70.6% of their active time feeding), resulting in irreversible and progressive dental tissue loss [8]. This directly impairs the core feeding function, generating a linked negative cascade of “oral health deterioration–feeding impairment–diminished quality of life”, which represents a critical and non-neglectable concern in the welfare management of both captive and wild giant pandas [9,10].

From our clinical experience, elderly captive pandas with advanced wear actively avoid hard bamboo culms, exhibit significantly shortened feeding durations, and present disrupted feeding frequencies; some individuals even display food refusal and stress-related behavioral abnormalities (e.g., agitation) presumably associated with chewing pain. Therefore, timely oral intervention and dietary adaptation are essential components of giant panda health and welfare management. However, quantitative data on giant panda tooth wear are remarkably scarce. Early studies focused on fossil specimens and morphological descriptions [2,11,12]. Researchers have described the gradual trend of premolar molarization, expansion of the molar occlusal surface, and development of nodular structures that enhance grinding efficiency.

Computer simulation of tooth wear has been successfully applied in veterinary dentistry to predict occlusal surface changes and pathological wear [13]. Applying similar methods to giant pandas could provide valuable insights for captive management. Therefore, we developed a two-dimensional simulation of giant panda tooth wear with the following objectives: (1) to quantify wear rates of canine, second premolar, and second molar teeth under horizontal vs. vertical bamboo chewing; (2) to compare wear resistance among tooth types; (3) to calibrate simulation cycles to real age using published wear criteria; and (4) to provide evidence-based recommendations for dental health management in captive giant pandas.

## 2. Materials and Methods

### 2.1. Baseline Data Acquisition of Giant Panda Teeth

With approval from the China Conservation and Research Center for the Giant Panda (Approval number CCRCGP2024004), serial cross-sectional computed tomography (CT) images of a giant panda skull were acquired during a routine physical examination. The subject was an 18-year-old adult male giant panda. Given that giant pandas primarily employ their canines, premolars, and molars during bamboo consumption [14,15], biomechanical analysis of bamboo chewing was performed specifically on these three tooth types. Among them, the second premolar and second molar are the main functional cheek teeth with the largest occlusal contact area during bamboo mastication in giant pandas. The first premolar is degenerated and tiny with negligible grinding function, while the third molar is underdeveloped in most individuals. Therefore, the second premolar and second molar were selected as representative teeth of premolar and molar groups to reflect the main wear characteristics of cheek teeth in the present study.

### 2.2. Tooth Model Simulation

The simulation was built in Processing (version 3.5.4, Processing Foundation, USA), an open-source Java-based graphical framework, which was modified in a previous study [13]. The occlusal shapes were modeled as irregular pixel matrices: canines have a rounded cusp, premolars have three rounded cusps and an asymmetrical occlusion, and molars have a central fossa and elevated marginal ridges.

The hardness of giant panda teeth enamel and dentine were measured by indenting them with 344 HV (Vickers Hardness) and 70.4 HV, respectively [11,16]. The real hardness was converted to simulation hardness using a 4:1 scaling factor for easy calculation, yielding enamel = 86 units and dentine = 17.6 units, consistent with a previous equine tooth wear simulation model [13]. Each pixel had a maximum damage threshold equal to the material hardness. A random damage factor (0.5% probability per cycle) simulated occasional hard objects (e.g., small stones) in bamboo.

### 2.3. Chewing Cycle and Bamboo Orientation

**Figure 1:**
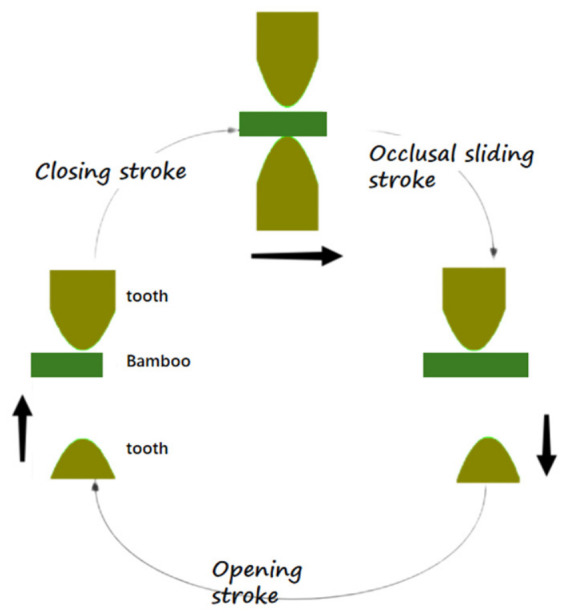
Scheme of simulated giant panda canine horizontal chewing cycles.

The chewing cycle comprised three phases—opening stroke, closing stroke, and power stroke—following the standard mammalian mastication kinematic model [13,17]. The counterclockwise reversal every three cycles was set to mimic the asymmetric lateral jaw movement observed in giant panda feeding videos (Supplementary Video S1), which avoids unidirectional single-sided tooth wear and restores natural random chewing postures. Two bamboo orientations were simulated separately: horizontal (bamboo stem perpendicular to tooth long axis) and vertical (bamboo stem parallel to tooth long axis). The giant pandas exhibited a preference for small bamboo species, including *B. fargesii*, *P. japonica*, and *I. tessellatus* [18]. In this study, bamboo was modeled as a cylinder of 15 mm diameter (150 pixel layers) with uniform hardness. Bite forces were applied perpendicular to the occlusal surface: 1030 N for canines, 1142 N for second premolars, and 2005 N for second molars [11,19,20] (Figure 1).

### 2.4. Wear Algorithm and Quantification

Wear was calculated using the Reye–Archard–Khrushchov wear law (volume loss ∝ sliding distance × normal force) [21]. During the power stroke, each contacting pixel accumulated damage. When cumulative damage exceeded the material-specific threshold, the pixel was removed, exposing the underlying pixel. The simulation recorded the cumulative number of pixel layers lost from the upper tooth after each chewing cycle.

Wear was measured as cumulative pixel layers lost at 25, 50, 75, and 100 cycles. Each condition (tooth type × chewing orientation) was simulated with the built-in random damage (0.5%) to provide minor stochastic variation.

### 2.5. Calibration to Real Age

According to a previous study, the molar cusps of giant pandas become flattened into a single plane at approximately 20–26 years of age [22]. Based on this finding, the number of wear cycles required to reach an equivalent level of molar flattening, estimated to correspond to 23 years of age, was defined as the terminal criterion in our simulation model. Subsequently, this model was employed to predict the actual wear times required to achieve analogous wear patterns on other tooth types.

## 3. Results

### 3.1. Morphometric Data of Giant Panda Teeth

**Figure 2:**
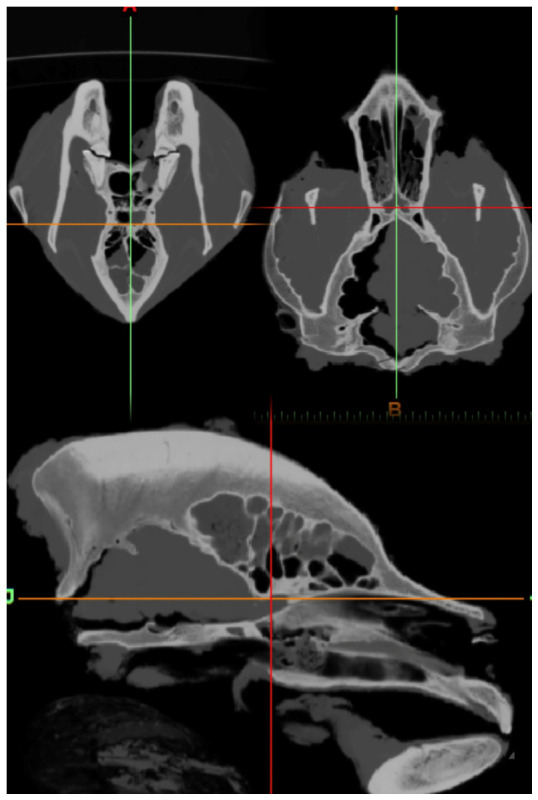
CT scan data of the giant panda.

The baseline morphometric data of the three tooth types (canine, second premolar, and second molar) used in the simulation are presented in Table 1. As shown in Figure 2, tooth dimensions were derived from CT scans of an 18-year-old adult male giant panda and converted to pixel units for simulation purposes. Maxillary canines were the tallest teeth (22.1 mm, 221 pixel layers), while mandibular second premolars were the shortest (5.5 mm, 55 pixel layers). Maxillary second molars exhibited the greatest width (36.6 mm, 366 pixel layers). Accordingly, the simulated model was shaped to match the geometry of the giant panda’s teeth.

### 3.2. Wear Patterns and Rates of Canine Tooth

**Figure 3:**
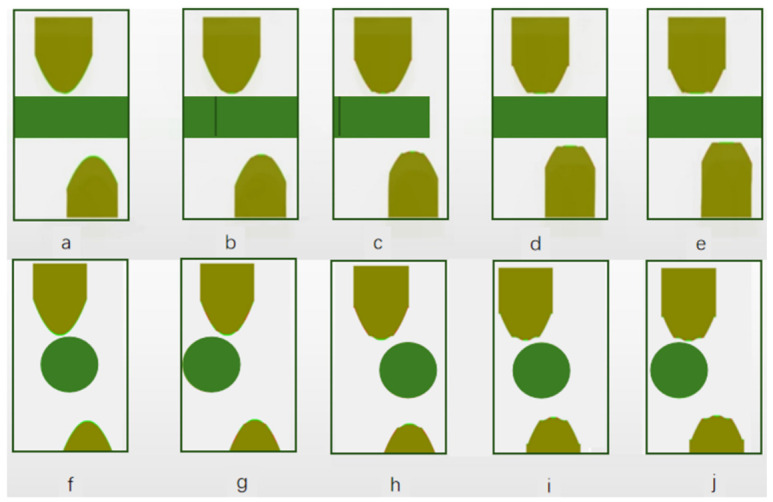
Friction model of canine teeth wear cycles ((**a**–**e**) bamboo horizontal: 1, 25, 50, 75, 100 cycles; (**f**–**j**) bamboo vertical: 1, 25, 50, 75, 100 cycles).

Canine wear was strongly influenced by chewing orientation (Figure 3). The simulation results demonstrated that after 75 chewing cycles, a wear plateau on the occlusal surface became visible in the normal direction for the horizontal bamboo chewing condition, with a worn layer thickness of 36.12 pixel layers. After 100 chewing cycles, the worn layer thickness increased to 45.68 pixel layers, and this value continued to increase during subsequent simulation cycles. For the vertical bamboo chewing condition, wear on the occlusal surface in the normal direction became visible after 100 chewing cycles, with a worn layer thickness of 36.76 pixel layers, which also increased progressively during further simulation cycles.

### 3.3. Wear Patterns and Rates of Premolar Teeth

Premolar wear was greater during vertical chewing (bamboo parallel to tooth long axis) than during horizontal chewing (Table 2). After 50 cycles, cusp wear was visible on the premolars, accompanied by a reduction in sulcus depth, corresponding to a wear thickness of 21.66 pixel layers. After 100 cycles, vertical chewing had removed 34.24 pixel layers, whereas horizontal chewing removed only 10.28 pixel layers. Vertical chewing rapidly reduced cusp height and shallowed the sulci, with visible wear beginning as early as 50 cycles, and both cusp wear and sulcus shallowing continued to progress during further simulation cycles. This orientation dependence indicates that premolars are primarily stressed when bamboo is aligned with the tooth row.

### 3.4. Wear Patterns and Rates of Molar Teeth

The wear pattern for molars mirrored that of the premolars, showing greater wear during vertical chewing (Table 2). Specifically, after 50 cycles, the depth of the molar central fossa was visibly reduced, with a measured wear of 20.96 pixel layers. After 100 cycles, vertical chewing resulted in the removal of 51.52 pixel layers, while horizontal chewing removed 39.40 pixel layers. Consequently, the central fossa became progressively more shallow, and by 100 cycles, the occlusal surface became noticeably flattened. Notably, the highest wear rate among all tooth types and chewing orientations was observed in the molars during vertical chewing. This finding reflects the molars’ primary role in grinding fibrous bamboo.

### 3.5. Predicted Functional Lifespan

**Figure 4:**
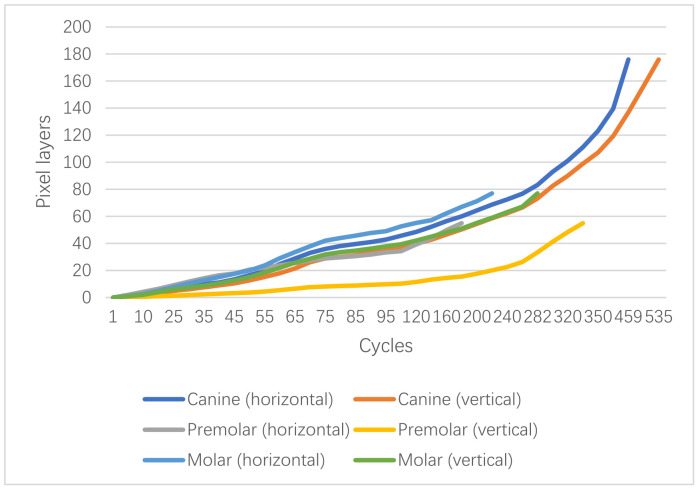
Wear and tear of canine teeth, premolars, and molars at different cycles.

In our simulation, complete tooth wear occurred between 179 and 535 cycles across all teeth (Figure 4). Among them, complete molar crown wear occurred at 206 cycles. The giant panda molar cusps are fully flattened at approximately 23 years old [22]. We found that the simulation reaches the same flat occlusal morphology at 206 chewing cycles. The conversion coefficient 23 years/206 cycles ≈ 38 days per cycle was only used for relative age conversion to predict functional tooth lifespan. Therefore, it assumed 1 cycle ≈ 38 days of real wear (23 years of age for 206 cycles). This calibration allowed for extrapolation from cycles to years for complete crown loss. Using the calibration, we extrapolated the number of cycles required for complete crown loss for each tooth type and chewing orientation. As shown in Table 3, premolars and molars are predicted to lose function by approximately 19–23 years under natural chewing conditions, whereas canines may last beyond 45 years. Among all configurations, the shortest functional lifespan was predicted for premolars with vertical chewing (19 years), followed by molars with vertical chewing (23 years). Canines showed the greatest longevity, with vertical chewing yielding an estimated lifespan of 55 years and horizontal chewing yielding an estimated lifespan of 47 years.

These predictions highlight that cheek teeth (premolars and molars), especially under vertical chewing (the orientation that likely dominates natural bamboo processing), are the most vulnerable to early functional loss. In contrast, canines are remarkably durable and unlikely to be a limiting factor for survival within the typical captive lifespan (≈30 years).

## 4. Discussion

### 4.1. Research Background and Methodological Advantages

This study presents the first quantitative, cycle-by-cycle computer simulation of tooth wear in giant pandas, integrating species-specific morphological, material, and biomechanical parameters. Our results provide novel insights into differential wear rates among tooth types, the influence of chewing orientation, and predicted functional lifespan. These findings have direct implications for dental health management in captive giant pandas in several aspects.

Common methods for analyzing mammalian masticatory motion typically include invasive animal experiments, such as three-dimensional X-ray reconstruction of kinematic morphology or intensive feeding trials [23,24]. For example, Weng et al. used preserved giant panda tooth specimens from the last century to conduct sectioning and indentation tests, demonstrating that giant panda enamel hardness exceeds that of human enamel [11], which provided a theoretical foundation for the present study. However, due to the scarcity of giant pandas, conducting research using giant panda tooth specimens is extremely difficult. Therefore, this study selected simulation software to test the wear resistance of different giant panda teeth. Using this method to test the wear resistance of giant panda teeth offers the following advantages: (1) it avoids destructive research on giant panda teeth; (2) this study was able to not only evaluate wear resistance among different tooth types but also identify vulnerable wear-prone areas, enabling preventive restoration of these locations in giant pandas; (3) additionally, the model can be optimized in future studies by selecting tooth wear morphologies at different cycle counts as templates for different stages of physiological or pathological wear, thereby providing a theoretical basis for future giant panda wear evaluation systems; and (4) this study incorporated random cycles into the chewing cycle to simulate the unconscious random chewing behavior exhibited by giant pandas during feeding, and therefore better simulated the masticatory trajectory of giant pandas.

The methodology was modified from Sterkenburgh et al. [13], who developed a 2D computer simulation of equine cheek tooth wear. Similar to their findings, we observed progressive occlusal flattening and sulcus shallowing with increasing cycles. However, important biological differences exist. Horses possess hypsodont (high-crowned) teeth that erupt continuously throughout life, partially compensating for occlusal wear. In contrast, giant pandas have brachydont (low-crowned) teeth with limited eruption potential. Consequently, wear in pandas is essentially irreversible and leads to permanent functional loss once crown height is exhausted. This difference underscores the greater importance of preventive dental management in pandas compared to equids. In the current standardized simulation model, bamboo was simplified as a uniform hard cylindrical material to control single variables (only chewing orientation and tooth type were compared). We clearly acknowledged that bamboo species, stem/leaf hardness differences were not incorporated into this two-dimensional model, and we propose that future research can add gradient bamboo hardness parameters to optimize the simulation system.

### 4.2. Biological Implications of Differential Wear Rates

Our simulated wear progression patterns are broadly consistent with earlier descriptive studies. Tikka et al. [14] observed that giant panda molars possess a broad, nodular occlusal surface that enhances grinding efficiency but undergoes progressive flattening with age. Our simulation reproduced this phenomenon: after 206 cycles (calibrated to approximately 23 years), molar cusps became nearly planar, matching the age-related occlusal flattening described by Wei et al. [22]. Similarly, Qigao et al. [3] noted progressive premolar molarization in older individuals, a change we observed as cusp fusion and sulcus shallowing under continued vertical chewing. Of note, compared to these qualitative descriptions, our simulation provides quantitative estimates of wear progression, predicting the number of cycles required for specific morphological changes. Visible premolar cusp height reduction began at approximately 50 cycles (≈5 years), a threshold not previously reported.

However, our prediction that premolars reach functional exhaustion earlier than molars should be interpreted with caution as it is based solely on mechanical wear rates and does not account for other failure modes such as fracture, caries, or periodontal disease. Clinical monitoring programs should therefore assess both tooth types regularly, with premolars warranting early attention (starting at ~10 years) based on our wear predictions, though molars may present different clinical challenges.

### 4.3. Clinical Management Applications and Recommendations

With progressive wear, occlusal surface tensile stress shifted from being concentrated in sulcal regions to becoming more evenly distributed [25]. This is a natural adaptive change that reduces the risk of crown failure in older individuals. This finding parallels our observation that premolar and molar wear rates tend to stabilize at later cycles (75–100 cycles, as shown in Table 2), potentially reflecting similar adaptive stress redistribution. More recently, Harty et al. [26] reported that increased herbivorous food intake causes severe enamel and dentin wear in Grauer’s gorillas and western lowland gorillas, which supports our conclusion that abrasive bamboo accelerates cheek tooth wear in giant pandas. This directly supports our findings that the abrasive bamboo diet of giant pandas drives rapid wear of cheek teeth. Additionally, the significant influence of chewing orientation on wear rates had not been previously quantified. Canines wore faster during horizontal chewing (45.68 pixel layers/100 cycles) compared to vertical chewing (36.76 pixel layers/100 cycles), whereas premolars and molars exhibited faster wear during vertical chewing (34.24 and 51.52 pixel layers/100 cycles, respectively) compared to horizontal chewing (10.28 and 39.40 pixel layers/100 cycles). This orientation-dependent wear can be explained by contact mechanics: horizontal bamboo creates point-contact on the canine tip, generating high localized pressure; vertical bamboo produces line-contact along the occlusal surface of cheek teeth, distributing the force but increasing the sliding distance [27]. These findings have practical implications. In captive settings, individual pandas exhibit variable bamboo handling behaviors. Some split bamboo lengthwise with canines (vertical orientation), while others crush it transversely (horizontal orientation). Our results suggest, in some special over wear cases, encouraging vertical chewing on canines (e.g., by providing pre-split bamboo) might reduce canine wear, and encouraging horizontal chewing on cheek teeth (by offering shorter segments) might reduce molar and premolar wear. However, modifying natural chewing behavior is challenging.

For the clinical relevance, our simulation provides a quantitative framework for predicting tooth wear progression in captive giant pandas. The clinical relevance of our results are as follows: (1) age-based dental monitoring should begin at 10 years (after 100 cycles, all tooth types showed marked wear); (2) special attention should be given to premolars as the shortest-lived teeth (19 years); and (3) individualized management should be based on observed chewing behavior, where feasible. By integrating dental health into routine health assessments, wildlife managers can improve the longevity and welfare of giant pandas under human care. This is particularly important given that wild giant panda populations face multiple health challenges [28].

### 4.4. Limitations and Future Research Directions

However, several limitations should be acknowledged. First, the simulation is two-dimensional and cannot fully capture the three-dimensional complexity of tooth morphology, occlusal contacts, and chewing kinematics. Second, it should be noted that this study is a preliminary investigation based on a single individual and idealized simulations of occlusion and tooth wear. We assumed uniform material properties for enamel and dentine, whereas enamel hardness may vary across tooth regions and with age. Future studies should validate our predictions using micro-CT of known-age captive pandas, develop three-dimensional wear simulations, incorporate individual variation in bite force and chewing behavior, and apply similar approaches to other captive wildlife consuming abrasive diets. Third, this study represents the first quantitative simulation of tooth wear in giant pandas; therefore, our predictions should be considered preliminary and require further validation with independent samples and empirical data. We envision that future work—incorporating additional individuals, broader age ranges, three-dimensional modeling approaches, and refined dietary parameters—will iteratively improve the model and enhance the generalizability of our findings. Fourth, we did not independently evaluate wear differences between maxillary and mandibular teeth in the present simulation. Our two-dimensional model primarily focused on occlusal contact kinematics rather than on the detailed crown morphology of each dental arch. Given that maxillary and mandibular teeth differ in cusp shape, root support, and occlusal contact angles, these anatomical variations may influence wear patterns in vivo. Future three-dimensional simulations with separate parameterization of upper and lower dentitions are warranted to address this question and will be incorporated into our subsequent model development work. Fifth, the calibration between simulation cycles and chronological age was based on a single age–wear reference point, which we acknowledge as a simplification. In reality, tooth wear rates in giant pandas may vary considerably depending on dietary composition and individual behavioral habits. Future studies incorporating a broader range of age cohorts and corresponding in vivo wear measurements are required to establish a more robust and generalizable calibration curve.

Our predictions are strictly applicable to adult pandas (≥5 years) with fully erupted permanent dentition. Juveniles will require separate parameterization of bite force, dietary composition, and tooth morphology. For female pandas, we recommend applying a correction factor of approximately 0.85–0.90 to the wear rate estimates presented here, based on the expected bite force reduction. However, given the uncertainty in this scaling, we suggest that a conservative (unadjusted) schedule be used for monitoring purposes, with individualized adjustments as clinical data accumulate. We emphasized repeatedly that all wear data are derived from a two-dimensional simulation model based on one single individual’s tooth morphology. The predicted functional lifespan only reflects relative wear trends, and cannot be directly used as absolute clinical age standards for all giant pandas. We suggest that zoo veterinarians combine actual individual age, feeding habits and oral examination results for comprehensive dental evaluation, instead of relying solely on this simulation prediction. All management suggestions are proposed as reference strategies rather than universal clinical standards.

## 5. Conclusions

The simulation predicts that premolars and molars will lose complete grinding function after the equivalent of 19–23 years of simulated chewing cycles, while canines maintain wear resistance far beyond this age range. These findings provide a quantitative basis for dental health management in captive giant pandas, including regular age-based monitoring, dietary texture modification, and early intervention for high-risk individuals. By integrating dental health into routine health assessments, wildlife managers can improve the longevity and welfare of giant pandas under human care. Previous studies have demonstrated that dietary composition can substantially influence tooth wear patterns. However, as a preliminary/proof-of-concept study, our current model remains relatively simplified and is not yet capable of simulating species-specific or individual-specific dietary variations. Our current model does not directly simulate dietary interventions but we have considered how it could be extended. Although such extensions would require considerable funding and time, we plan to collaborate with multiple institutions in the future to further refine this model, with the goal of providing more tailored recommendations for giant panda and wildlife management.

## Figures and Tables

**Table 1:** Morphometric data of giant panda teeth used in the simulation.

Tooth	Arch	Width (mm)	Width (Pixel Layers)	Height (mm)	Height (Pixel Layers)
Canine	Maxillary	17.6	176	22.1	221
Canine	Mandibular	12.6	126	17.6	176
Second premolar	Maxillary	19.3	193	7.4	74
Second premolar	Mandibular	11.6	116	5.5	55
Second molar	Maxillary	36.6	366	8.4	84
Second molar	Mandibular	27.9	279	7.7	77

**Table 2:** Cumulative pixel layers lost after 25, 50, 75, and 100 chewing cycles.

Tooth	Orientation	25 Cycles	50 Cycles	75 Cycles	100 Cycles
Canine	Horizontal	5.60	17.36	35.88	45.68
Canine	Vertical	4.84	13.40	28.88	36.76
Premolar	Horizontal	1.30	3.88	8.10	10.28
Premolar	Vertical	9.08	21.66	28.96	34.24
Molar	Horizontal	6.04	16.00	31.88	39.40
Molar	Vertical	7.72	20.96	42.00	51.52

**Table 3:** Predicted functional lifespan (complete crown loss) by tooth type and chewing orientation.

Tooth	Orientation	Cycles to Complete Wear	Estimated Age (Years)
Canine	Horizontal	459	47
Canine	Vertical	535	55
Premolar	Horizontal	327	34
Premolar	Vertical	179	19
Molar	Horizontal	282	29
Molar	Vertical	220	23

## Data Availability

The original contributions presented in the study are included in the article; further inquiries can be directed to the corresponding authors.

## Supplementary Materials

The following supporting information can be downloaded at: https://www.mdpi.com/article/10.3390/ani16142273/s1, Figure S1: Friction model of premolar and molar teeth wear; Video S1: A giant panda eating bamboo. Supplementary S1: The original code for the simulation.
