# Supplementary material for: Computer Simulation of Tooth Wear in Giant Pandas (Ailuropoda melanoleuca): Quantitative Predictions for Captive Health Management
Source: Animals (Basel). 2026 Jul 22;16(14):2273. doi: 10.3390/ani16142273 (PMC13403947; doi:10.3390/ani16142273)
Supplement: Supplementary file 1 [file animals-16-02273-s001.zip › Figure S1. Friction model of premolar and molar teeth wear.pdf]

Figure S1 Friction model of premolar and molar teeth wear;

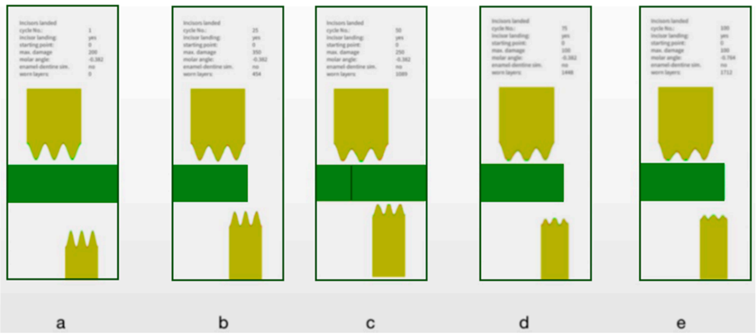

Friction model of premolar teeth (bamboo biting vertically)

Note: upper and lower premolars underwent a:1 wear cycle; b: 25 wear cycles; c: 50 wear cycles; d: 75 wear cycles; e: 100 wear cycles

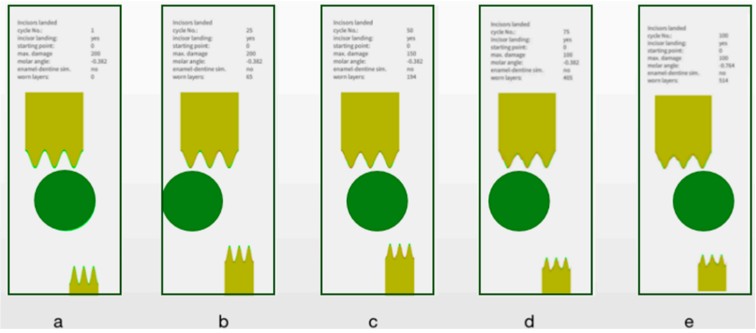

Friction model of premolar teeth (bamboo biting horizontally)

Note: upper and lower premolars underwent a:1 wear cycle; b: 25 wear cycles; c: 50 wear cycles; d: 75 wear cycles; e: 100 wear cycles

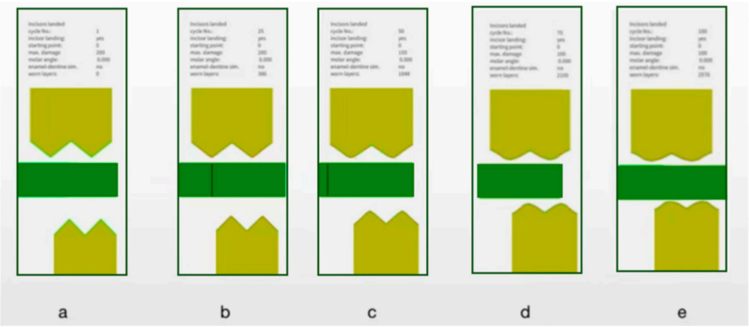

Friction model of molar teeth (bamboo biting vertically)

Note: upper and lower molars underwent a:1 wear cycle; b: 25 wear cycles; c: 50 wear cycles; d: 75 wear cycles; e: 100 wear cycles

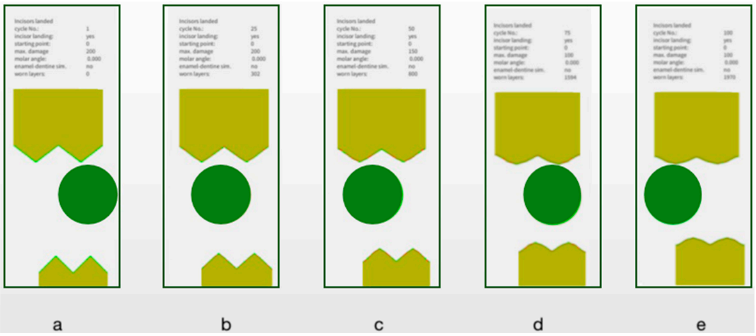

Friction model of molar teeth (bamboo biting horizontally)

Note: upper and lower molars underwent a:1 wear cycle; b: 25 wear cycles; c: 50 wear cycles; d: 75 wear cycles; e: 100 wear cycles
