# Supplementary material for: Computer Simulation of Tooth Wear in Giant Pandas (Ailuropoda melanoleuca): Quantitative Predictions for Captive Health Management
Source: Animals (Basel). 2026 Jul 22;16(14):2273. doi: 10.3390/ani16142273 (PMC13403947; doi:10.3390/ani16142273)
Supplement: Supplementary file 1 [file animals-16-02273-s001.zip › Supplementary S1.pdf]

Supplementary S1: The original code for the simulation.

Canine:

```
class Tooth {
	PVector pos;
	PVector move;

	int[][] toothStructure;
	final int YVALUE = 0;
	final int DAMAGE = 1;
	final int STRUCTURE_PRESET = 2;
	final int MAX_DAMAGE = 350;
	final boolean TOOTH_BODY_VISUALIZATION = true;
	final boolean SIMULATE_DENTINE = true;
	int toothWidth;
	int heightMolar;
	boolean maxilla;
	int colorChannel;
	int wornLayers;
float bambooX = 0;
int bambooDirection = 1;

	Tooth(PVector pos, int toothWidth, int heightMolar, boolean maxilla) {
		this.pos = pos;
		this.toothWidth = toothWidth;
		this.maxilla = maxilla;
		this.heightMolar = heightMolar;
		toothStructure = new int[toothWidth][3];
		move = new PVector(0, 0);
		wornLayers = 0;
		for (int i = 0; i < toothWidth; i++) {
			float waveValue = calculateCanineShape(i, toothWidth);
			toothStructure[i][YVALUE] = (int) (waveValue * heightMolar / 2);
			toothStructure[i][DAMAGE] = 0;
			toothStructure[i][STRUCTURE_PRESET] = (int) (15 * MAX_DAMAGE / 20);
		}
	}

	float calculateCanineShape(int i, int toothWidth) {
		float x = (float) i / (float) (toothWidth - 1);
		float y = map(abs(0.5 - x), 0, 0.5, 1, 0);
		y = pow(1 - y, 2);
		return y;
	}
```

```
    void render() {
        pushMatrix();
        translate(width / 3 - 50, height * 2 / 3 - 275);
        for (int i = 0; i < toothWidth; i++) {
            if (toothStructure[i][DAMAGE] >= 0) {
                colorChannel = 255 - 250 * toothStructure[i][DAMAGE] / MAX_DAMAGE;
                int colorValue = toothStructure[i][STRUCTURE_PRESET] * 180 /
MAX_DAMAGE;

                stroke(colorValue, colorValue, 0);
                if (maxilla) {
                    line(pos.x + i, pos.y, pos.x + i, pos.y + heightMolar -
toothStructure[i][YVALUE]);
                    stroke(255 - colorChannel, colorChannel, 0);
                    line(pos.x + i, pos.y + heightMolar - toothStructure[i][YVALUE],
                         pos.x + i, pos.y + heightMolar - 2 - toothStructure[i][YVALUE]);
                } else {
                    line(pos.x + i, pos.y + heightMolar, pos.x + i, pos.y + 2 +
toothStructure[i][YVALUE]);
                    stroke(255 - colorChannel, colorChannel, 0);
                    line(pos.x + i, pos.y + 2 + toothStructure[i][YVALUE],
                         pos.x + i, pos.y + toothStructure[i][YVALUE]);
                }
            }
        }
        popMatrix();
    }

    void updatePosition() {
        pos.add(move);
    }

    void applyDamage(int x, int y) {
        int index = x;
        if (index >= 0 && index < toothWidth) {
            if (toothStructure[index][this.YVALUE] > 0) {
                if (toothStructure[index][DAMAGE] < MAX_DAMAGE) {
                    toothStructure[index][DAMAGE]++;
                } else {
                    toothStructure[index][this.YVALUE]--;
                    toothStructure[index][DAMAGE] = 0;
                    wornLayers++;
                }
```

```
                if (toothStructure[index][this.YVALUE] <= 0) {
                    toothStructure[index][this.YVALUE] = 0;
                }
            }

            int oppositeIndex = toothWidth - 1 - index;
            if (toothStructure[oppositeIndex][this.YVALUE] > 0) {
                if (toothStructure[oppositeIndex][DAMAGE] < MAX_DAMAGE) {
                    toothStructure[oppositeIndex][DAMAGE]++;
                } else {
                    toothStructure[oppositeIndex][this.YVALUE]--;
                    toothStructure[oppositeIndex][DAMAGE] = 0;
                    wornLayers++;
                }
                if (toothStructure[oppositeIndex][this.YVALUE] <= 0) {
                    toothStructure[oppositeIndex][this.YVALUE] = 0;
                }
            }
        }
    }
}

final int POWERSTROKE = 1;
final int OPENINGSTROKE = 2;
final int CLOSINGSTROKE = 3;
final int INCISORTOUCH = 4;

int strokeState = CLOSINGSTROKE;
int startContactLine, endContactLine;
int neutral_position_x;
int i_Lower;
int delta_pos_x;
float bambooX = 0;
int bambooDirection = 1;
final int WIDTH_MOLAR = 150;
final int HEIGHT_MOLAR = 200;

int powerStrokeStartPoint = 0;
final boolean SIMULATE_INCISOR_LANDING = true;
final float INCISOR_TOUCH_START_POINT = 0.50;

Tooth upperTooth, lowerTooth;
boolean in_touch;
int cycle;
```

```
int directionCount = 0;

void setup() {
	size(300, 750);
	initSimulation();
	colorMode(RGB, 255);
}

void initSimulation() {
	upperTooth = new Tooth(new PVector(0, 0), WIDTH_MOLAR, HEIGHT_MOLAR, true);
	lowerTooth = new Tooth(new PVector(0, HEIGHT_MOLAR + 4 * (WIDTH_MOLAR / 3)),
WIDTH_MOLAR, HEIGHT_MOLAR, false);
	neutral_position_x = upperTooth.toothWidth - int(float(lowerTooth.toothWidth) *
INCISOR_TOUCH_START_POINT);
	lowerTooth.move.x = 1;
	lowerTooth.move.y = 0;
	cycle = 1;
}


void textOutput() {
	pushMatrix();
	translate(20, 20);
	textSize(16);
	fill(60);
	switch (strokeState) {
		case POWERSTROKE:
			text("Power Stroke", 10, 30);
			break;
		case CLOSINGSTROKE:
			text("Closing Stroke", 10, 30);
			break;
		case OPENINGSTROKE:
			text("Opening Stroke", 10, 30);
			break;
		case INCISORTOUCH:
			text("Incisors landed", 10, 30);
			break;
	}
	text("cycle No.:", 10, 50);
	text(cycle, 200, 50);
	text("incisor landing:", 10, 70);
	text(SIMULATE_INCISOR_LANDING ? "yes" : "no", 200, 70);
	text("starting point:", 10, 90);
```

```
	text(powerStrokeStartPoint, 200, 90);
	text("max. damage", 10, 110);
	text(lowerTooth.MAX_DAMAGE, 200, 110);
	text("molar angle:", 10, 130);
	text(degrees(atan(float(upperTooth.toothStructure[WIDTH_MOLAR - 1][0]
	- upperTooth.toothStructure[0][0]) /
float(WIDTH_MOLAR))), 200, 130);
	text("enamel-dentine sim.", 10, 150);
	text(upperTooth.SIMULATE_DENTINE ? "yes" : "no", 200, 150);
	text("worn layers:", 10, 170);
	text(upperTooth.wornLayers, 200, 170);   // 输出 upperTooth 的 wornLayers
	popMatrix();
}

void draw() {
	background(240);
	noFill();
	textOutput();
	lowerTooth.render();
	upperTooth.render();

bambooX += int(bambooDirection * 2);

if (bambooX >1.5* WIDTH_MOLAR   || bambooX < 0) {
	bambooDirection *= -1;
}
	fill(0, 255, 0);
	rectMode(CORNERS);
	int bambooHeight = WIDTH_MOLAR / 3;
	int bambooWidth = int(1.5* WIDTH_MOLAR);
	int bambooY = HEIGHT_MOLAR + (int)(2.5 * bambooHeight) + (int)(1.2 * bambooHeight)
+ (1*bambooHeight);
	rect(-WIDTH_MOLAR / 2, bambooY, bambooWidth - WIDTH_MOLAR / 2, bambooY +
bambooHeight);
	rect(bambooX - WIDTH_MOLAR / 2, bambooY, bambooX + bambooWidth -
WIDTH_MOLAR / 2, bambooY + bambooHeight);

	if (lowerTooth.pos.x > bambooX - WIDTH_MOLAR / 2 && lowerTooth.pos.x < bambooX
+ bambooWidth - WIDTH_MOLAR / 2 &&
		lowerTooth.pos.y >= bambooY && lowerTooth.pos.y <= bambooY +
bambooHeight) {
		lowerTooth.applyDamage(lowerTooth.toothWidth - 1, 0);
	}
```

```
        if (upperTooth.pos.x > bambooX - WIDTH_MOLAR / 2 && upperTooth.pos.x < bambooX
+ bambooWidth - WIDTH_MOLAR / 2 &&
                upperTooth.pos.y >= bambooY && upperTooth.pos.y <= bambooY +
bambooHeight) {
                upperTooth.applyDamage(0, 0);
        }
        if ((lowerTooth.pos.x > neutral_position_x) &&
                ((strokeState == INCISORTOUCH) || !SIMULATE_INCISOR_LANDING)) {
                strokeState = OPENINGSTROKE;
                cycle++;
                lowerTooth.move.x = -1;
                lowerTooth.move.y = 1;
                if ((cycle == 2 || cycle == 25 || cycle == 50 || cycle == 75 || cycle == 100 || cycle ==
200) ) {
                        saveFrame("sim_far_" + cycle + ".png");                 }
        }

        if ((lowerTooth.pos.x <= powerStrokeStartPoint) &&
                (lowerTooth.pos.y > HEIGHT_MOLAR + 15 - upperTooth.toothStructure[1][0] -
lowerTooth.toothStructure[1][0])) {
                strokeState = CLOSINGSTROKE;
                lowerTooth.move.y = -1;
                lowerTooth.move.x = 0;

    }

        if ((lowerTooth.pos.x <= powerStrokeStartPoint) &&
                (lowerTooth.pos.y <= HEIGHT_MOLAR+ (int)( 1.2* bambooHeight))) {
                strokeState = POWERSTROKE;



        }




if ((lowerTooth.pos.x > upperTooth.toothWidth * INCISOR_TOUCH_START_POINT) &&
        (strokeState == POWERSTROKE) && SIMULATE_INCISOR_LANDING) {
        strokeState = INCISORTOUCH;
        lowerTooth.move.x = 1;
        lowerTooth.move.y = 0;
}

if (strokeState == POWERSTROKE) {
        lowerTooth.move.x = 2;
```

```
		lowerTooth.move.y = 4;
		in_touch = false;
		startContactLine = max(int(upperTooth.pos.x), int(lowerTooth.pos.x));
		endContactLine = min(int(lowerTooth.pos.x) + lowerTooth.toothWidth,
		                     int(upperTooth.pos.x) + upperTooth.toothWidth);
		delta_pos_x = int(upperTooth.pos.x) - int(lowerTooth.pos.x);
		for (int i = startContactLine; i < endContactLine; i++) {
			i_Lower = i + delta_pos_x;
			if ((lowerTooth.pos.y - upperTooth.pos.y - upperTooth.toothStructure[i][0] - lowerTooth.toothStructure[i_Lower][0]) < HEIGHT_MOLAR+ (int)( 1.2* bambooHeight)) {
				lowerTooth.applyDamage(i_Lower, 0);
				upperTooth.applyDamage(i, 0);
				in_touch = true;
			}
		}
		if (!in_touch) {
			lowerTooth.move.x = 0;
			lowerTooth.move.y = -1;
		}
	}

	if (strokeState == OPENINGSTROKE) {
		lowerTooth.move.x = -1;
		lowerTooth.move.y = 1;
		in_touch = false;
	}

	if (strokeState == CLOSINGSTROKE) {
		lowerTooth.move.x = 0;
		lowerTooth.move.y = -1;
		in_touch = false;
	}

	upperTooth.updatePosition();
	lowerTooth.updatePosition();

	if (cycle % 5 == 0 && directionCount < 2) {
		lowerTooth.move.y = -1;
		directionCount++;
	} else {
		lowerTooth.move.y = 1;
		if (directionCount >= 2) {
			directionCount = 0;
		}
```

```
}
}
```

Premolar：

```
class Tooth {
        PVector pos;
        PVector move;

        int[][] toothStructure;
    final int YVALUE = 0;
    final int DAMAGE = 1;
    final int STRUCTURE_PRESET = 2;
    final int MAX_DAMAGE = 350;
    final boolean TOOTH_BODY_VISUALIZATION = true;
    final boolean SIMULATE_DENTINE = false;
    final float[] structure = {4, 5, 6, 8, 10, 13, 15, 17, 20, 22, 20, 17, 15, 13, 10, 8, 6, 5, 4}; // canine
tooth shape
    int toothWidth;
    int heightMolar;
    boolean maxilla;
    int colorChannel;
    int wornLayers;
    float bambooX = 0;
int bambooDirection = 1;
    Tooth(PVector pos, int toothWidth, int heightMolar, boolean maxilla) {
        this.pos = pos;
        this.toothWidth = toothWidth;
        this.maxilla = maxilla;
        this.heightMolar = heightMolar;
        toothStructure = new int[toothWidth][3];
        move = new PVector(0, 0);

                generateBiteSurface();
        }
      void generateBiteSurface() {
        final int BASE_HEIGHT = 50;
        final int EDGE_HEIGHT = 5;
        final float SMOOTHNESS = 0.4;
```

```
	for (int i = 0; i < toothWidth; i++) {
		if (i < toothWidth / 6) {
			toothStructure[i][YVALUE] = int(BASE_HEIGHT - (BASE_HEIGHT - 
EDGE_HEIGHT) * smoothStep(i, 0, toothWidth / 6));
		} else if (i < 2 * toothWidth / 6) {
			toothStructure[i][YVALUE] = int(EDGE_HEIGHT + (BASE_HEIGHT - 
EDGE_HEIGHT) * smoothStep(i - toothWidth / 6, 0, toothWidth / 6));
		} else if (i < 3 * toothWidth / 6) {
			toothStructure[i][YVALUE] =int( BASE_HEIGHT - (BASE_HEIGHT - 
EDGE_HEIGHT) * smoothStep(i - 2 * toothWidth / 6, 0, toothWidth / 6));
		} else if (i < 4 * toothWidth / 6) {
			toothStructure[i][YVALUE] = int(EDGE_HEIGHT + (BASE_HEIGHT - 
EDGE_HEIGHT) * smoothStep(i - 3 * toothWidth / 6, 0, toothWidth / 6));
		} else if (i < 5 * toothWidth / 6) {
			toothStructure[i][YVALUE] = int(BASE_HEIGHT - (BASE_HEIGHT - 
EDGE_HEIGHT) * smoothStep(i - 4 * toothWidth / 6, 0, toothWidth / 6));
		} else {
			toothStructure[i][YVALUE] = int(EDGE_HEIGHT + (BASE_HEIGHT - 
EDGE_HEIGHT) * smoothStep(i - 5 * toothWidth / 6, 0, toothWidth / 6));
		}

		toothStructure[i][DAMAGE] = 0;
		toothStructure[i][STRUCTURE_PRESET] = SIMULATE_DENTINE ?
			int((toothStructure[i][YVALUE] + EDGE_HEIGHT) * MAX_DAMAGE / (2 * 
BASE_HEIGHT)) : MAX_DAMAGE;
	}
}

float smoothStep(float x, float min, float max) {
	x = constrain((x - min) / (max - min), 0, 1);
	return x * x * (3 - 2 * x);
}


	void render() {
		pushMatrix();
		translate(width / 3 - 50, height * 2 / 3 - 275);
		for (int i = 0; i < toothWidth; i++) {
			if (toothStructure[i][DAMAGE] >= 0) {
				colorChannel = 255 - 250 * toothStructure[i][DAMAGE] / MAX_DAMAGE;
				int colorValue = toothStructure[i][STRUCTURE_PRESET] * 180 / 
MAX_DAMAGE;

				stroke(colorValue, colorValue, 0);
```

```
                if (maxilla) {
                    line(pos.x + i, pos.y, pos.x + i, pos.y + heightMolar - toothStructure[i][YVALUE]);
                    stroke(255 - colorChannel, colorChannel, 0);
                    line(pos.x + i, pos.y + heightMolar - toothStructure[i][YVALUE],
                         pos.x + i, pos.y + heightMolar - 2 - toothStructure[i][YVALUE]);
                } else {
                    line(pos.x + i, pos.y + heightMolar, pos.x + i, pos.y + 2 + toothStructure[i][YVALUE]);
                    stroke(255 - colorChannel, colorChannel, 0);
                    line(pos.x + i, pos.y + 2 + toothStructure[i][YVALUE],
                         pos.x + i, pos.y + toothStructure[i][YVALUE]);
                }
            }
        }
        popMatrix();
    }

    void updatePosition() {
        pos.add(move);
    }
void applyDamage(int x, int y) {
    int index = x;
    if (index >= 0 && index < toothWidth) {
        if (toothStructure[index][YVALUE] > 0) {
            if (toothStructure[index][DAMAGE] < MAX_DAMAGE) {
                toothStructure[index][DAMAGE]++;
            } else {
                toothStructure[index][YVALUE]--;
                toothStructure[index][DAMAGE] = 0;
                wornLayers++;
            }
            if (toothStructure[index][YVALUE] <= 0) {
                toothStructure[index][YVALUE] = 0;
            }
        }

        int oppositeIndex = index + 1;
        if (oppositeIndex < toothWidth && toothStructure[oppositeIndex][YVALUE] > 0) {
            if (toothStructure[oppositeIndex][DAMAGE] < MAX_DAMAGE) {
                toothStructure[oppositeIndex][DAMAGE]++;
            } else {
                toothStructure[oppositeIndex][YVALUE]--;
                toothStructure[oppositeIndex][DAMAGE] = 0;
```

```
                    wornLayers++;
                }
                if (toothStructure[oppositeIndex][YVALUE] <= 0) {
                    toothStructure[oppositeIndex][YVALUE] = 0;
                }
            }

            int upperIndex = toothWidth - 1 - index;
            if (upperIndex >= 0 && upperIndex < toothWidth) {
                if (toothStructure[upperIndex][YVALUE] > 0) {
                    if (toothStructure[upperIndex][DAMAGE] < MAX_DAMAGE) {
                        toothStructure[upperIndex][DAMAGE]++;
                    } else {
                        toothStructure[upperIndex][YVALUE]--;
                        toothStructure[upperIndex][DAMAGE] = 0;
                        wornLayers++;
                    }
                    if (toothStructure[upperIndex][YVALUE] <= 0) {
                        toothStructure[upperIndex][YVALUE] = 0;
                    }
                }
            }
        }
    }


}

final int POWERSTROKE = 1;
final int OPENINGSTROKE = 0;
final int CLOSINGSTROKE = 3;
final int INCISORTOUCH = 4;

int strokeState = POWERSTROKE;
int startContactLine, endContactLine;
int neutral_position_x;
int i_Lower;
int delta_pos_x;
float bambooX = 0;

int bambooDirection = 1;
final int WIDTH_MOLAR = 150;
final int HEIGHT_MOLAR = 200;
```

```
int powerStrokeStartPoint = 0;
final boolean SIMULATE_INCISOR_LANDING = true;
final float INCISOR_TOUCH_START_POINT = 0.50;

Tooth upperTooth, lowerTooth;
boolean in_touch;
int cycle;
int directionCount = 0;

void setup() {
	size(300, 750);
	initSimulation();
	colorMode(RGB, 255);
}

void initSimulation() {
	upperTooth = new Tooth(new PVector(0, 0), WIDTH_MOLAR, HEIGHT_MOLAR, true);
	lowerTooth = new Tooth(new PVector(0, HEIGHT_MOLAR + 4 * (WIDTH_MOLAR / 3)),
WIDTH_MOLAR/2, HEIGHT_MOLAR, false);
	neutral_position_x = upperTooth.toothWidth - int(float(lowerTooth.toothWidth) *
INCISOR_TOUCH_START_POINT);
	lowerTooth.move.x = 1;
	lowerTooth.move.y = 0;
	cycle = 1;
}


void textOutput() {
	pushMatrix();
	translate(20, 20);
	textSize(16);
	fill(60);
	switch (strokeState) {
		case POWERSTROKE:
			text("Power Stroke", 10, 30);
			break;
		case CLOSINGSTROKE:
			text("Closing Stroke", 10, 30);
			break;
		case OPENINGSTROKE:
			text("Opening Stroke", 10, 30);
			break;
		case INCISORTOUCH:
			text("Incisors landed", 10, 30);
```

```
			break;
		}
		text("cycle No.:", 10, 50);
		text(cycle, 200, 50);
		text("incisor landing:", 10, 70);
		text(SIMULATE_INCISOR_LANDING ? "yes" : "no", 200, 70);
		text("starting point:", 10, 90);
		text(powerStrokeStartPoint, 200, 90);
		text("max. damage", 10, 110);
		text(lowerTooth.MAX_DAMAGE, 200, 110);
		text("molar angle:", 10, 130);
		text(degrees(atan(float(upperTooth.toothStructure[WIDTH_MOLAR - 1][0]
							-		upperTooth.toothStructure[0][0])		/
float(WIDTH_MOLAR))), 200, 130);
		text("enamel-dentine sim.", 10, 150);
		text(upperTooth.SIMULATE_DENTINE ? "yes" : "no", 200, 150);
		text("worn layers:", 10, 170);
		text(upperTooth.wornLayers, 200, 170);   // 输出 upperTooth 的 wornLayers

		popMatrix();
}

void draw() {
		background(240);		noFill();
		textOutput();
		lowerTooth.render();
		upperTooth.render();
bambooX += int(bambooDirection * 2);

if (bambooX >1.5* WIDTH_MOLAR   || bambooX < 0) {
		bambooDirection *= -1;
}
		fill(0, 255, 0);
		rectMode(CORNERS);
		int bambooHeight = WIDTH_MOLAR / 3;
		int bambooWidth = int(1.5* WIDTH_MOLAR);
		int bambooY = HEIGHT_MOLAR + (int)(2.5 * bambooHeight) + (int)(1.2 * bambooHeight)
+ (1*bambooHeight);
		rect(-WIDTH_MOLAR / 2, bambooY, bambooWidth - WIDTH_MOLAR / 2, bambooY +
bambooHeight);
		rect(bambooX - WIDTH_MOLAR / 2, bambooY, bambooX + bambooWidth -
WIDTH_MOLAR / 2, bambooY + bambooHeight);

		if (lowerTooth.pos.x > bambooX - WIDTH_MOLAR / 2 && lowerTooth.pos.x < bambooX
```

```
+ bambooWidth - WIDTH_MOLAR / 2 &&
            lowerTooth.pos.y >= bambooY && lowerTooth.pos.y <= bambooY + bambooHeight) {
            lowerTooth.applyDamage(lowerTooth.toothWidth - 1, 0);
      }

      if (upperTooth.pos.x > bambooX - WIDTH_MOLAR / 2 && upperTooth.pos.x < bambooX + bambooWidth - WIDTH_MOLAR / 2 &&
            upperTooth.pos.y >= bambooY && upperTooth.pos.y <= bambooY + bambooHeight) {
            upperTooth.applyDamage(0, 0);
      }
      if ((lowerTooth.pos.x > neutral_position_x) &&
            ((strokeState == INCISORTOUCH) || !SIMULATE_INCISOR_LANDING)) {
            strokeState = OPENINGSTROKE;
            cycle++;
            lowerTooth.move.x = -1;
            lowerTooth.move.y = 1;
            if ((cycle == 2 || cycle == 25 || cycle == 50 || cycle == 75 || cycle == 100 || cycle == 200) ) {
                  saveFrame("sim_far_" + cycle + ".png");
            }
      }

      if ((lowerTooth.pos.x <= powerStrokeStartPoint) &&
            (lowerTooth.pos.y > HEIGHT_MOLAR + 15 - upperTooth.toothStructure[1][0] - lowerTooth.toothStructure[1][0])) {
            strokeState = CLOSINGSTROKE;
            lowerTooth.move.y = -1;
            lowerTooth.move.x = 0;

   }

      if ((lowerTooth.pos.x <= powerStrokeStartPoint) &&
            (lowerTooth.pos.y <= HEIGHT_MOLAR+ (int)( 1.2* bambooHeight))) {
            strokeState = POWERSTROKE;



      }
```

```
if ((lowerTooth.pos.x > upperTooth.toothWidth * INCISOR_TOUCH_START_POINT) &&
        (strokeState == POWERSTROKE) && SIMULATE_INCISOR_LANDING) {
        strokeState = INCISORTOUCH;
        lowerTooth.move.x = 1;
        lowerTooth.move.y = 0;
}

if (strokeState == POWERSTROKE) {
        lowerTooth.move.x = 2;
        lowerTooth.move.y = 4;
        in_touch = false;
        startContactLine = max(int(upperTooth.pos.x), int(lowerTooth.pos.x));
        endContactLine = min(int(lowerTooth.pos.x) + lowerTooth.toothWidth,
                                        int(upperTooth.pos.x) + upperTooth.toothWidth);
        delta_pos_x = int(upperTooth.pos.x) - int(lowerTooth.pos.x);
        for (int i = startContactLine; i < endContactLine; i++) {
                i_Lower = i + delta_pos_x;
                if   ((lowerTooth.pos.y   -   upperTooth.pos.y   -   upperTooth.toothStructure[i][0]   -
lowerTooth.toothStructure[i_Lower][0]) < HEIGHT_MOLAR+ (int)( 1.2* bambooHeight)) {
                        lowerTooth.applyDamage(i_Lower, 0);
                        upperTooth.applyDamage(i, 0);
                        in_touch = true;
                }
        }
        if (!in_touch) {
                lowerTooth.move.x = 0;
                lowerTooth.move.y = -1;
        }
}

if (strokeState == OPENINGSTROKE) {
        lowerTooth.move.x = -1;
        lowerTooth.move.y = 1;
        in_touch = false;
}

if (strokeState == CLOSINGSTROKE) {
        lowerTooth.move.x = 0;
        lowerTooth.move.y = -1;
        in_touch = false;
}

upperTooth.updatePosition();
lowerTooth.updatePosition();
```

```
if (cycle % 5 == 0 && directionCount < 2) {
        lowerTooth.move.y = -1;
        directionCount++;
} else {
        lowerTooth.move.y = 1;
        if (directionCount >= 2) {
                directionCount = 0;
        }
}
}
```

Molar:

```
class Tooth {
        PVector pos;
        PVector move;

        int[][] toothStructure;
    final int YVALUE = 0;
    final int DAMAGE = 1;
    final int STRUCTURE_PRESET = 2;
    final int MAX_DAMAGE = 350;
    final boolean TOOTH_BODY_VISUALIZATION = true;
    final boolean SIMULATE_DENTINE = false;
    final float[] structure = {4, 5, 6, 8, 10, 13, 15, 17, 20, 22, 20, 17, 15, 13, 10, 8, 6, 5, 4}; // canine
tooth shape
    int toothWidth;
    int heightMolar;
    boolean maxilla;
    int colorChannel;
    int wornLayers;
```

```
   float bambooX = 0;
int bambooDirection = 1;

   Tooth(PVector pos, int toothWidth, int heightMolar, boolean maxilla) {
      this.pos = pos;
      this.toothWidth = toothWidth;
      this.maxilla = maxilla;
      this.heightMolar = heightMolar;
      toothStructure = new int[toothWidth][3];
      move = new PVector(0, 0);
      wornLayers = 0;
final int BASE_HEIGHT = 50; /
final int EDGE_HEIGHT = 5;

for (int i = 0; i < toothWidth; i++) {
      float distanceFromCenter = abs(i - toothWidth / 2.0);
      toothStructure[i][YVALUE] = int(map(distanceFromCenter, 0, toothWidth / 2.0,
BASE_HEIGHT, EDGE_HEIGHT));
      toothStructure[i][DAMAGE] = 0;
      toothStructure[i][STRUCTURE_PRESET] = SIMULATE_DENTINE ? int(structure[int(i *
float(structure.length) / toothWidth)] * MAX_DAMAGE / 22) : MAX_DAMAGE;
}
   }

      float calculateCanineShape(int i, int toothWidth) {
         float x = (float) i / (float) (toothWidth - 1);
         float y = map(abs(0.5 - x), 0, 0.5, 1, 0);
         y = pow(1 - y, 2);
         return y;
      }

      void render() {
         pushMatrix();
         translate(width / 3 - 50, height * 2 / 3 - 275);
         for (int i = 0; i < toothWidth; i++) {
            if (toothStructure[i][DAMAGE] >= 0) {
               colorChannel = 255 - 250 * toothStructure[i][DAMAGE] / MAX_DAMAGE;
               int colorValue = toothStructure[i][STRUCTURE_PRESET] * 180 /
MAX_DAMAGE;

               stroke(colorValue, colorValue, 0);
               if (maxilla) {
                  line(pos.x + i, pos.y, pos.x + i, pos.y + heightMolar -
toothStructure[i][YVALUE]);
```

```
                    stroke(255 - colorChannel, colorChannel, 0);
                    line(pos.x + i, pos.y + heightMolar - toothStructure[i][YVALUE],
                         pos.x + i, pos.y + heightMolar - 2 - toothStructure[i][YVALUE]);
                } else {
                    line(pos.x + i, pos.y + heightMolar, pos.x + i, pos.y + 2 +
toothStructure[i][YVALUE]);
                    stroke(255 - colorChannel, colorChannel, 0);
                    line(pos.x + i, pos.y + 2 + toothStructure[i][YVALUE],
                         pos.x + i, pos.y + toothStructure[i][YVALUE]);
                }
            }
        }
        popMatrix();
    }

    void updatePosition() {
        pos.add(move);
    }

    void applyDamage(int x, int y) {
        int index = x;
        if (index >= 0 && index < toothWidth) {
            if (toothStructure[index][this.YVALUE] > 0) {
                if (toothStructure[index][DAMAGE] < MAX_DAMAGE) {
                    toothStructure[index][DAMAGE]++;
                } else {
                    toothStructure[index][this.YVALUE]--;
                    toothStructure[index][DAMAGE] = 0;
                    wornLayers++;
                }
                if (toothStructure[index][this.YVALUE] <= 0) {
                    toothStructure[index][this.YVALUE] = 0;
                }
            }

            int oppositeIndex = toothWidth - 1 - index;
            if (toothStructure[oppositeIndex][this.YVALUE] > 0) {
                if (toothStructure[oppositeIndex][DAMAGE] < MAX_DAMAGE) {
                    toothStructure[oppositeIndex][DAMAGE]++;
                } else {
                    toothStructure[oppositeIndex][this.YVALUE]--;
                    toothStructure[oppositeIndex][DAMAGE] = 0;
                    wornLayers++;
                }
```

```
                    if (toothStructure[oppositeIndex][this.YVALUE] <= 0) {
                        toothStructure[oppositeIndex][this.YVALUE] = 0;
                    }
                }
            }
        }
}

final int POWERSTROKE = 1;
final int OPENINGSTROKE = 2;
final int CLOSINGSTROKE = 3;
final int INCISORTOUCH = 4;

int strokeState = POWERSTROKE;
int startContactLine, endContactLine;
int neutral_position_x;
int i_Lower;
int delta_pos_x;
float bambooX = 0;

int bambooDirection = 1;
final int WIDTH_MOLAR = 150;
final int HEIGHT_MOLAR = 200;

int powerStrokeStartPoint = 0;
final boolean SIMULATE_INCISOR_LANDING = true;
final float INCISOR_TOUCH_START_POINT = 0.50;

Tooth upperTooth, lowerTooth;
boolean in_touch;
int cycle;
int directionCount = 0;

void setup() {
    size(300, 750);
    initSimulation();
    colorMode(RGB, 255);
}

void initSimulation() {
    upperTooth = new Tooth(new PVector(0, 0), WIDTH_MOLAR, HEIGHT_MOLAR, true);
    lowerTooth = new Tooth(new PVector(0, HEIGHT_MOLAR + 4 * (WIDTH_MOLAR / 3)),
WIDTH_MOLAR, HEIGHT_MOLAR, false);
    neutral_position_x = upperTooth.toothWidth - int(float(lowerTooth.toothWidth) *
```

```
INCISOR_TOUCH_START_POINT);
        lowerTooth.move.x = 1;
        lowerTooth.move.y = 0;
        cycle = 1;
}


void textOutput() {
        pushMatrix();
        translate(20, 20);
        textSize(16);
        fill(60);
        switch (strokeState) {
                case POWERSTROKE:
                        text("Power Stroke", 10, 30);
                        break;
                case CLOSINGSTROKE:
                        text("Closing Stroke", 10, 30);
                        break;
                case OPENINGSTROKE:
                        text("Opening Stroke", 10, 30);
                        break;
                case INCISORTOUCH:
                        text("Incisors landed", 10, 30);
                        break;
        }
        text("cycle No.:", 10, 50);
        text(cycle, 200, 50);
        text("incisor landing:", 10, 70);
        text(SIMULATE_INCISOR_LANDING ? "yes" : "no", 200, 70);
        text("starting point:", 10, 90);
        text(powerStrokeStartPoint, 200, 90);
        text("max. damage", 10, 110);
        text(lowerTooth.MAX_DAMAGE, 200, 110);
        text("molar angle:", 10, 130);
        text(degrees(atan(float(upperTooth.toothStructure[WIDTH_MOLAR - 1][0]
                                          -               upperTooth.toothStructure[0][0])               /
float(WIDTH_MOLAR))), 200, 130);
        text("enamel-dentine sim.", 10, 150);
        text(upperTooth.SIMULATE_DENTINE ? "yes" : "no", 200, 150);
        text("worn layers:", 10, 170);
        text(upperTooth.wornLayers, 200, 170);   // 输出 upperTooth 的 wornLayers

        popMatrix();
```

```
}

void draw() {
	background(240);
	noFill();
	textOutput();
	lowerTooth.render();
	upperTooth.render();
bambooX += int(bambooDirection * 2);

if (bambooX >1.5* WIDTH_MOLAR	|| bambooX < 0) {
	bambooDirection *= -1;
}
	fill(0, 255, 0);
	rectMode(CORNERS);
	int bambooHeight = WIDTH_MOLAR / 3;
	int bambooWidth = int(1.5* WIDTH_MOLAR);
	int bambooY = HEIGHT_MOLAR + (int)(2.5 * bambooHeight) + (int)(1.2 * bambooHeight)
+ (1*bambooHeight);
	rect(-WIDTH_MOLAR / 2, bambooY, bambooWidth - WIDTH_MOLAR / 2, bambooY +
bambooHeight);
	rect(bambooX - WIDTH_MOLAR / 2, bambooY, bambooX + bambooWidth -
WIDTH_MOLAR / 2, bambooY + bambooHeight);

	if (lowerTooth.pos.x > bambooX - WIDTH_MOLAR / 2 && lowerTooth.pos.x < bambooX
+ bambooWidth - WIDTH_MOLAR / 2 &&
		lowerTooth.pos.y >= bambooY && lowerTooth.pos.y <= bambooY +
bambooHeight) {
		lowerTooth.applyDamage(lowerTooth.toothWidth - 1, 0);
	}

	if (upperTooth.pos.x > bambooX - WIDTH_MOLAR / 2 && upperTooth.pos.x < bambooX
+ bambooWidth - WIDTH_MOLAR / 2 &&
		upperTooth.pos.y >= bambooY && upperTooth.pos.y <= bambooY +
bambooHeight) {
		upperTooth.applyDamage(0, 0);
	}
	if ((lowerTooth.pos.x > neutral_position_x) &&
		((strokeState == INCISORTOUCH) || !SIMULATE_INCISOR_LANDING)) {
		strokeState = OPENINGSTROKE;
		cycle++;
		lowerTooth.move.x = -1;
		lowerTooth.move.y = 1;
		if ((cycle == 2 || cycle == 25 || cycle == 50 || cycle == 75 || cycle == 100 || cycle ==
```

```
200) ) {
                saveFrame("sim_far_" + cycle + ".png");
            }
        }

        if ((lowerTooth.pos.x <= powerStrokeStartPoint) &&
            (lowerTooth.pos.y > HEIGHT_MOLAR + 15 - upperTooth.toothStructure[1][0] -
lowerTooth.toothStructure[1][0])) {
            strokeState = CLOSINGSTROKE;
            lowerTooth.move.y = -1;
            lowerTooth.move.x = 0;

      }

        if ((lowerTooth.pos.x <= powerStrokeStartPoint) &&
            (lowerTooth.pos.y <= HEIGHT_MOLAR+ (int)( 1.2* bambooHeight))) {
            strokeState = POWERSTROKE;


        }



if ((lowerTooth.pos.x > upperTooth.toothWidth * INCISOR_TOUCH_START_POINT) &&
        (strokeState == POWERSTROKE) && SIMULATE_INCISOR_LANDING) {
        strokeState = INCISORTOUCH;
        lowerTooth.move.x = 1;
        lowerTooth.move.y = 0;
}

if (strokeState == POWERSTROKE) {
        lowerTooth.move.x = 2;
        lowerTooth.move.y = 4;
        in_touch = false;
        startContactLine = max(int(upperTooth.pos.x), int(lowerTooth.pos.x));
        endContactLine = min(int(lowerTooth.pos.x) + lowerTooth.toothWidth,
                             int(upperTooth.pos.x) + upperTooth.toothWidth);
        delta_pos_x = int(upperTooth.pos.x) - int(lowerTooth.pos.x);
        for (int i = startContactLine; i < endContactLine; i++) {
            i_Lower = i + delta_pos_x;
            if ((lowerTooth.pos.y - upperTooth.pos.y - upperTooth.toothStructure[i][0] -
lowerTooth.toothStructure[i_Lower][0]) < HEIGHT_MOLAR+ (int)( 1.2* bambooHeight)) {
                lowerTooth.applyDamage(i_Lower, 0);
                upperTooth.applyDamage(i, 0);
```

```
                in_touch = true;
            }
        }
        if (!in_touch) {
            lowerTooth.move.x = 0;
            lowerTooth.move.y = -1;
        }
    }

    if (strokeState == OPENINGSTROKE) {
        lowerTooth.move.x = -1;
        lowerTooth.move.y = 1;
        in_touch = false;
    }

    if (strokeState == CLOSINGSTROKE) {
        lowerTooth.move.x = 0;
        lowerTooth.move.y = -1;
        in_touch = false;
    }

    upperTooth.updatePosition();
    lowerTooth.updatePosition();

    if (cycle % 5 == 0 && directionCount < 2) {
        lowerTooth.move.y = -1;
        directionCount++;
    } else {
        lowerTooth.move.y = 1;
        if (directionCount >= 2) {
            directionCount = 0;
        }
    }
    }
```
